# Severe pelvic injury: vascular lesions detected by ante- and post-mortem contrast medium-enhanced CT and associations with pelvic fractures

**DOI:** 10.1007/s00414-016-1503-4

**Published:** 2016-11-28

**Authors:** Mahmoud Hussami, Silke Grabherr, Reto A. Meuli, Sabine Schmidt

**Affiliations:** 1 Department of Diagnostic and Interventional Radiology, University Hospital of Lausanne, Rue du Bugnon 46, 1011 Lausanne, Switzerland; 2 University Center of Legal Medicine Lausanne—Geneva, University of Lausanne, Rue du Bugnon 46, 1011 Lausanne, Switzerland

**Keywords:** Multi-phase post-mortem CT-angiography (MPMCTA), Multi-detector computed tomography (MDCT), Vascular system injuries, Pelvic bone fractures, Pelvic fracture bleeding, Forensic radiology

## Abstract

**Objectives:**

The objectives of this study were to compare arterial and venous contrast medium extravasation in severe pelvic injury detected by ante- and post-mortem multi-detector CT (MDCT) and determine whether vascular injury is associated with certain types of pelvic fracture.

**Methods:**

We retrospectively included two different cohorts of blunt pelvic trauma with contrast medium extravasation shown by MDCT. The first group comprised 49 polytrauma patients; the second included 45 dead bodies undergoing multi-phase post-mortem CT-angiography (MPMCTA). Two radiologists jointly reviewed each examination concerning type, site of bleeding and pattern of underlying pelvic ring fracture.

**Results:**

All 49 polytrauma patients demonstrated arterial bleeding, immediately undergoing subsequent angiography; 42 (85%) had pelvic fractures, but no venous bleeding was disclosed. MPMCTA of 45 bodies revealed arterial (*n* = 33, 73%) and venous (*n* = 35, 78%) bleeding and pelvic fractures (*n* = 41, 91%). Pelvic fracture locations were significantly correlated with ten arterial and six venous bleeding sites in dead bodies, with five arterial bleeding sites in polytrauma patients.

In dead bodies, arterial haemorrhage was significantly correlated with the severity of pelvic fracture according to Tile classification (*p* = 0.01), unlike venous bleeding (*p* = 0.34).

**Conclusions:**

In severe pelvic injury, certain acute bleeding sites were significantly correlated with underlying pelvic fracture locations. MPMCTA revealed more venous lesions than MDCT in polytrauma patients. Future investigations should evaluate the proportional contribution of venous bleeding to overall pelvic haemorrhage as well as its clinical significance.

## Introduction

Pelvic fractures occur in 4–9.3% of patients with blunt trauma, and the prevalence of associated organ injuries ranges from 11 to 20.3%. [1, 2]. Pelvic haemorrhage is the most serious complication associated with pelvic fractures, and active haemorrhage remains the leading cause of death in polytrauma patients [2–4]. Massive pelvic haemorrhage may originate from the branches of the iliac artery and/or major pelvic veins, from the small arteries running within the fractured bone or from the pelvic venous plexus [1, 5–8].

In most emergency departments, polytrauma patients are initially evaluated by contrast medium-enhanced multi-detector computed tomography (MDCT) to detect active haemorrhage and enable immediate patient management and straightforward therapeutic decisions [2, 9, 10]. Detection of contrast medium extravasation on MDCT corresponds well to the site of bleeding seen on subsequent conventional angiography [1, 4, 9, 10] Furthermore, early detection of active bleeding by MDCT may lead to prompt angiographic embolisation. The latter has a technical success rate of up to 100%, with few complications, and it has been proven to be lifesaving [2, 4, 8, 11, 12]. Thus, immediate angiography and subsequent trans-catheter embolisation are currently accepted as the most effective methods for controlling arterial bleeding resulting from pelvic fractures [1, 8–10, 13–15]. However, little is known about the incidence and clinical significance of venous bleeding in these polytrauma patients. Moreover, no clinical series of polytrauma patients has directly correlated the bleeding sites with the underlying fractured pelvic bones.

The recent development of multi-phase post-mortem CT angiography (MPMCTA) has enabled the detection of vascular lesions in dead bodies, particularly those lesions present after severe trauma [16, 17]. Since the examination can be performed with a considerable volume of contrast agent injected at fast speed and with high pressure, MPMCT allows for the diagnosis of vessel injuries in great detail without disruption of nearby anatomical structures, unlike conventional autopsy. The technique was evaluated in a multi-centre study that included 500 autopsy cases, and it is the most widespread and best-investigated method for dead bodies [18].

Our objective in the present study was to investigate whether arterial or venous vascular lesions were responsible for contrast medium extravasation in blunt pelvic trauma victims. Furthermore, we explored whether the anatomical site of the vascular lesions corresponded with certain, well-defined patterns of pelvic fracture, since both are associated with the same kinetics due to underlying trauma.

## Materials and methods

We retrospectively included two different cohorts of severe blunt trauma victims who were referred to our hospital after an acute traffic accident, crush, or fall. They had all undergone contrast media-enhanced MDCT.

### Clinical MDCT acquisition

After entering the keywords “polytrauma”, “pelvic fracture”, “active bleeding” and “acute haemorrhage” in our comprehensive database of examination reports, we retrieved 141 polytrauma patients who were admitted to our emergency department from January 2002 to February 2014. Immediately after their arrival, these patients had been investigated with intravenous (IV) contrast-enhanced MDCT. We only included patients for whom active contrast medium extravasation of the pelvic vessels was described in the examination reports. We excluded children under 16 years, all MDCT performed after surgery or angiographic embolisation of pelvic haemorrhage and patients with extrapelvic haemorrhage only. Note that bleeding detected by MDCT was not constantly found by subsequent angiography. Nevertheless, the arteries from which extravasation were proven by MDCT were always embolised and the patients then did well.

From among 141 patients, we evaluated 64 patients who had undergone simultaneous conventional angiography with confirmation of vascular lesions detected by MDCT.

Among these 64 patients, we excluded 15 patients for the following reasons: In ten patients, we could not confirm the presence of active haemorrhage on MDCT at admission during our review of the images, three patients had undergone MDCT at a different hospital without transmission of their images, and another two patients underwent MDCT after treatment only (surgery and embolisation of pelvic haemorrhage). Thus, our final cohort comprised 49 polytrauma patients (15 women, mean age 51.9 years, age range 16–93 years).

Our polytrauma protocol was performed from January 2002 to November 2005 with a 16-detector row CT machine (Light Speed 16 Advantage; GE Healthcare, Milwaukee, USA) and from November 2005 to February 2014 with a 64-detector row CT machine (Light Speed VCT 64 Pro; GE Healthcare, Milwaukee, WI, USA). We acquired 1.25 mm reconstructed axial slices (increment of 1 mm) during the arterial phase (25 s) centred on the thorax and 2.5 mm reconstructed axial slices (increment of 2 mm) during the venous phase (80s) centred on the abdomen and pelvis, after IV injection of the iodinated contrast medium Accupaque®, (iohexol, 300 mgI/ml; volume in millilitres = body weight + 30 ml, GE Healthcare) at a flow rate of 4 ml/s (120 kV, 300 mA, table speed 55 mm per rotation (0.8 s), pitch 1.375). With the 64-detector row CT machine, we used automatic tube current modulation in all three axes (SmartmA) as well as the iterative reconstruction algorithm ASIR.

### Multi-phase post-mortem CT angiography acquisition

Since January 2009, our institute of legal medicine has performed MPMCTA on bodies that were referred to us for medicolegal reasons. Based on the institutional written report system, we selected all of those bodies admitted after severe blunt trauma (from traffic accident, crush or fall) between January 2009 and February 2014, in which active pelvic bleeding had been shown by MPMCTA. We excluded children under 16 years, any MPMCTA performed after surgical or radiological treatment of arterial bleeding and all cases with extrapelvic bleeding only.

Among 52 bodies in which active pelvic haemorrhage was identified, we excluded seven cases for the following reasons: In six of the bodies, pelvic haemorrhage was described on the radiological report but not confirmed during our review on the workstation and, in one case, there was an absent arterial phase due to a problem with femoral arterial cannulation. The final cohort comprised 45 post-mortem cases (15 women, mean age 53.1 years, age range 22–87 years).

All bodies included in this study were examined on a eight-detector row MDCT machine (GE Lightspeed, GE Healthcare, Milwaukee, WI, USA), using a field of view of 50 cm, and a reconstructed slice thickness of 1.2 mm (increment of 0.6 mm) for the arterial phase, 1.25 mm (increment of 1 mm) for the venous phase and 2.5 mm (increment of 2 mm) for the dynamic phase (120 kV, 300 mA, noise index 15, pitch 1.35 mm, rotation time 0.8 s).

For contrast media injection, arterial and venous femoral cannulas were connected to an extracorporeal perfusion device (Virtangio®Machine, Fumedica AG, Muri, Switzerland). Contrast media was composed of paraffin oil (paraffinum liquidum) and the iodised linseed oil Angiofil® (Fumedica AG), diluted to 6% (3.5 l paraffin oil with 210 ml of Angiofil) [16, 19]. The oily paraffin component is necessary to keep the contrast media within the vascular compartment of the corpse and to avoid extravasation into the surrounding interstitial tissue [16, 19]. Four different acquisitions were performed: unenhanced, arterial and venous phases followed by a dynamic phase. We started the arterial acquisition at 1.5 min after injecting 1200 ml of contrast agent mixture at a flow rate of 800 ml/min and the venous acquisition at 2.25 min after injecting 1800 ml of contrast agent mixture at a flow rate of 800 ml/min, which is 13.3 ml/s. The dynamic phase was acquired 70–80 s after reinjecting 500 ml of contrast medium at a flow rate of 200 ml/min (3.33 ml/s) and during an ongoing perfusion of the contrast agent through the vessels via an arterial injection. [16].

### Image analysis

In consensus, two radiologists (SaS and MH) with 15 and 4 years of practical experience in body imaging, respectively, reviewed all of the MDCT images of the polytrauma patients and the MPMCTA images of the bodies on an electronic workstation (Carestream Solutions, Carestream Health, Rochester, NY, USA). They were blinded to the results of previous reports, especially those concerning the presence and type of pelvic fracture as well as the presence and site of vascular injury. They registered the type of contrast medium a extravasation (arterial vs. venous) that occurred and the precise site of the vascular lesion. Active haemorrhage was defined as extravascular accumulation of contrast medium measuring >90 Hounsfield units (HU). Table 1 shows the arteries and veins we included in our image analysis. We analysed right and left vascular pelvic bleedings separately, as well as right and left pelvic fractures.

**Table 1:** Analysis of the different pelvic vessels and bones

Pelvic vessels (arteries and veins)	Common iliac, external iliac, internal iliac Posterior branches: iliolumbar, lateral sacral, superior gluteal Anterior branches: obturator, inferior gluteal, internal pudendal
Pelvic bones	Iliac wing, iliopubic branch, ischiopubic branch, acetabulum, sacral wing
Articulations	Sacroiliac joints, symphysis
Tile classification [20].	Stable pelvic ring fracture (Tile A), partial unstable pelvic ring fracture (Tile B), unstable pelvic ring fracture (Tile C)

The investigators also recorded the pattern and type of pelvic fracture, if any, according to the Tile classification of pelvic fractures [20–23] (Table 1).

### Statistical analysis

Statistical analysis was performed with the JMP 10 statistical package (SAS Institute, Inc., Cary, NC, USA). The presence and numbers of vascular lesions or pelvic fractures are expressed as categorical numbers and percentages. To determine the relationship between the site of any vascular bleeding and the type of fracture and between mechanism of trauma and site of vascular bleeding, we used Fisher’s exact test. Chi-square test was used to evaluate the relationship between any vascular lesion and the severity of pelvic ring fracture (Tile classification) and between the mechanism of trauma and Tile classification. All differences were considered significant at *p* < 0.05.

## Results

### Clinical findings

In our final cohort of 49 polytrauma patients, 24 (49%) were admitted after falls, 19 (39%) after traffic accidents and 6 (12%) out of them were victims of crush injuries, The mean time between MDCT and angiography was 124 min (median 60 min; min 15 min/max 24 h).

According to our inclusion criteria, all 49 polytrauma patients demonstrated at least one active haemorrhage on MDCT. Our image analysis revealed a total of 96 arterial lesions (average 5.8; median 6) without any venous lesions.

Forty-two (85%) of the 49 patients had pelvic fractures.

**Fig. 1:**
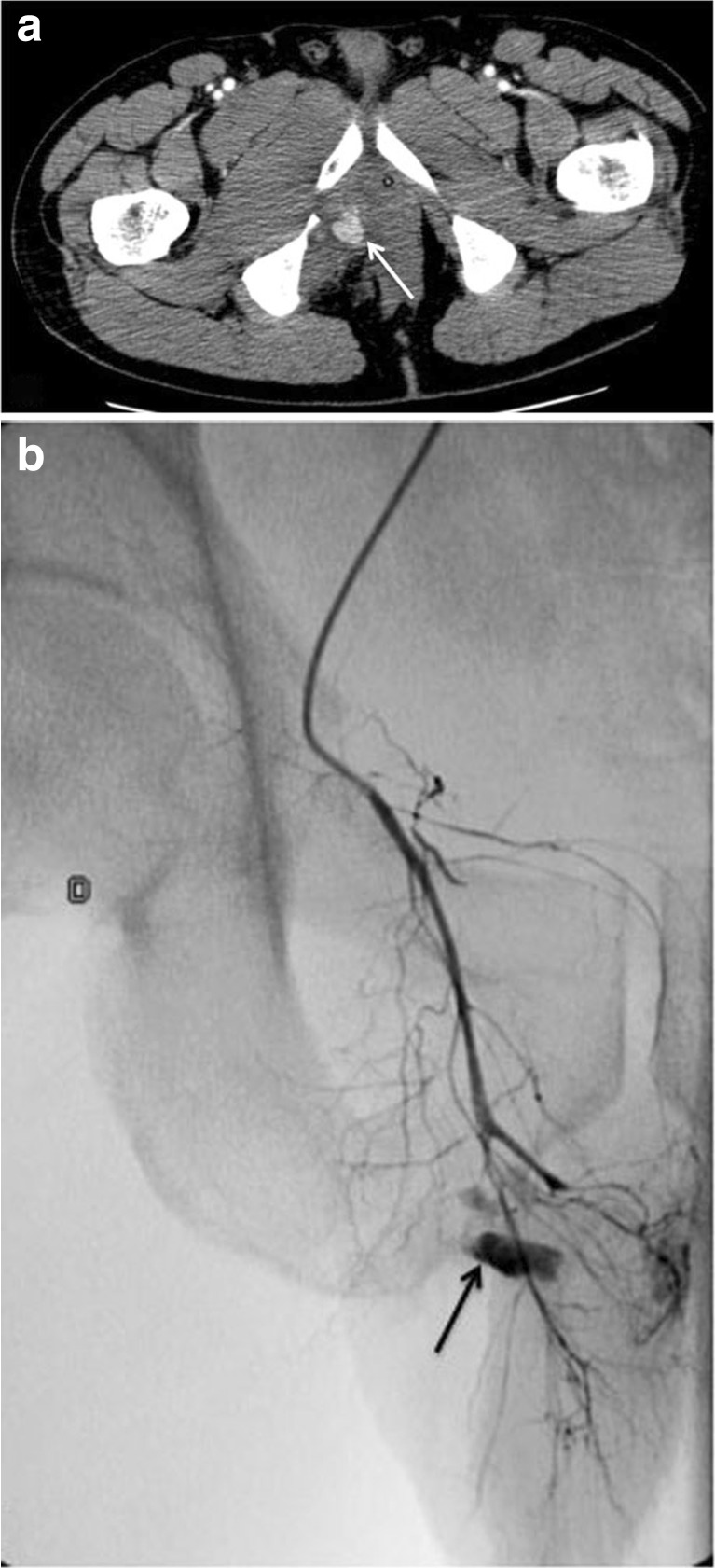
Clinical MDCT of a traffic accident victim. **a** Axial contrast-enhanced MDCT image shows right pudendal artery bleeding (*arrow*) associated with an ischiopubic branch fracture. **b** Arterial angiography performed immediately upon arrival confirmed the active bleeding from the right pudendal artery (*arrow*), and the patient was immediately treated by embolisation

The details about the most injured vessels and pelvic bone fractures are shown in Table 2 *.* Five arterial bleeding sites were significantly correlated with a pelvic fracture site (Table 3 and Fig. 1).

**Table 2:** Frequency of injuries in clinical examinations and post-mortem cases

	Clinical examinations (*n* = 49)	Post-mortem cases (*n* = 45)
Most injured arteries	Total injured arteries (*n* = 96)	Total injured arteries (*n* = 105)
	Superior gluteal (*n* = 22)	Obturator (*n* = 26)
	Lateral sacral (*n* = 21)	Iliolumbar (*n* = 22)
	Obturator (*n* = 20)	Lateral sacral (*n* = 15)
Most injured veins		Total injured veins (*n* = 79)
		Obturator (*n* = 13)
		Lateral sacral (*n* = 12)
		External iliac (*n* = 11)
Pelvic fractures	Total fractures (*n* = 173)	Total fractures (*n* = 195)
	Sacral wing (*n* = 43)	Sacral wing (*n* = 38)
	Ischiopubic (*n* = 38)	Ischiopubic (*n* = 38)
	Iliopubic (*n* = 34)	Iliopubic (*n* = 35)
	Acetabulum (*n* = 15)	Acetabulum (*n* = 31)
	Iliac wing (*n* = 14)	Iliac wing (*n* = 21)
	Symphysis disjunction (*n* = 15)	Symphysis disjunction (*n* = 14)
	Sacroiliac disjunction (*n* = 14)	Sacroiliac disjunction (*n* = 18)
Severity of fractures (Tile)		
Tile A	*n* = 24, 49%	*n* = 21, 47%
Tile B	*n* = 8, 16%	*n* = 6, 13%
Tile C	*n* = 10, 20%	*n* = 14, 31%

**Table 3:** Clinical examinations (polytrauma patients)—significant correlations between bleeding site and pelvic fracture

Pelvic arteries	Bone/articulation	*p* value
Left Lateral sacral artery	Left iliac wing	<0.05
Left superior gluteal artery	Left sacral wing	<0.05
Left superior gluteal artery	Left sacroiliac disjunction	<0.05
Left pudendal artery	Left ischiopubic branch	<0.05
Right obturator artery	Symphysis disjunction	<0.05

There was no significant correlation between the number of arterial bleeding sites detected per patient and the severity of pelvic ring fracture (Tile classification) (*p* > 0.05).

Our search for any statistically significant relation between the trauma mechanism and the bleeding site or the Tile classification only revealed one significant result: we detected more bleedings from the lateral sacral arteries in fall injuries (*p* < 0.05) than in patients admitted after “traffic accidents” or “crush” injuries. However, this was only true for the clinical examinations, i.e. the polytrauma patients, whereas in post-mortem cases, no statistically significant relation was found.

### Post-mortem findings

In our final cohort of 45 post-mortem cases, 31 (69%) victims died from a traffic accident, 11 (24%) from falls and, in 3 bodies (7%), the mechanism of injury were unknown.

The delay between death and MPMCTA varied from 24 to 72 h (average 35 h, median 24 h).

According to the inclusion criteria, all 45 bodies presented at least one active pelvic haemorrhage on MPMCTA. Thirty-three (73%) bodies demonstrated one or more arterial pelvic bleeding sites. Thirty-five (78%) bodies presented one or more venous pelvic bleeding sites.

**Fig. 2:**
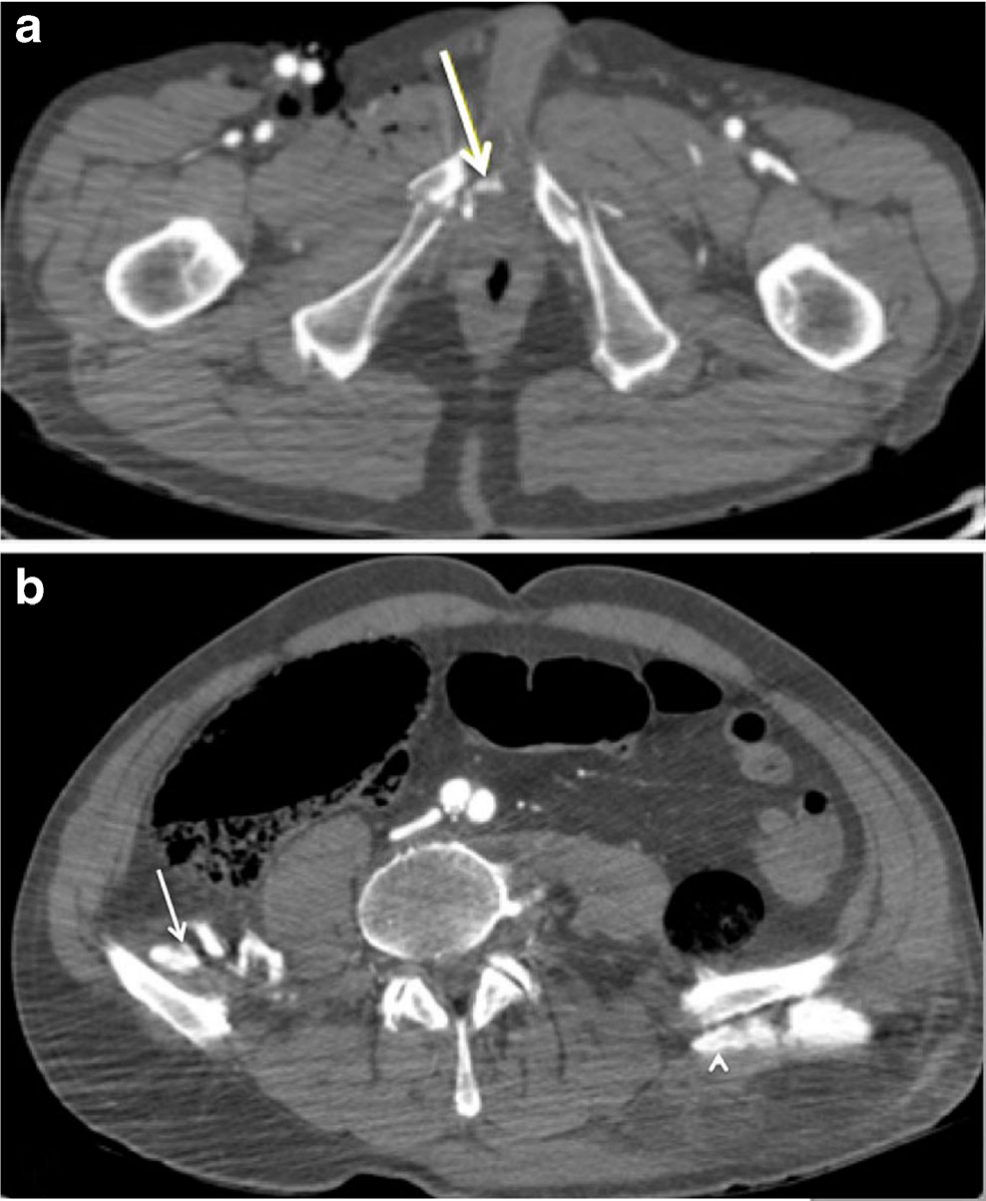
Multi-phase post-mortem CT-angiography (MPMCTA) of a victim after a fatal fall injury. **a** Axial MPMCTA image during arterial phase shows right pudendal artery bleeding (*arrow*) associated with bilateral ischiopubic rami fractures. **b** In the same cadaver, axial MPMCTA image acquired during dynamic phase demonstrates bleeding of the iliac branches of the right iliolumbar artery (*arrow*) and left superior gluteal artery (*arrowhead*) associated with bilateral iliac wing fractures (not shown)

**Fig. 3:**
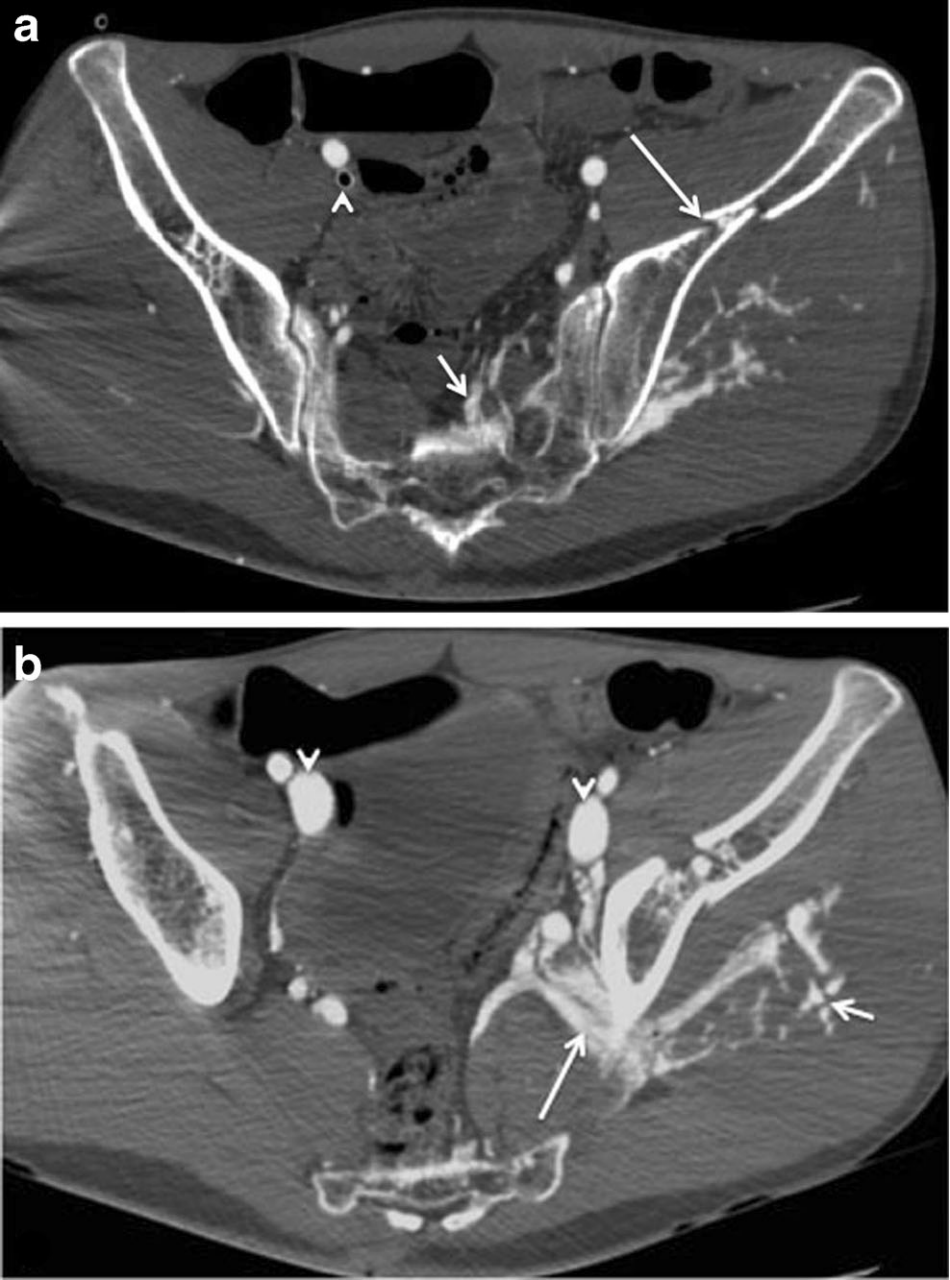
Multi-phase post-mortem CT-angiography (MPMCTA) of a victim after a fatal traffic accident. **a** Axial MPMCTA image acquired during arterial phase shows left lateral sacral artery bleeding (*short arrow*), left iliac wing (*long arrow*) and left sacral wing fractures (not shown). Note the wide cannula in the right external iliac vein (*arrowhead*), which is useful for administrating a large volume of contrast medium during the venous phase. **b** In the same cadaver, axial image acquired during venous phase demonstrates substantial extravasation of contrast agent from the left superior gluteal vein (*long arrow*) and from its superficial branches (*short arrow*). Note the contrast medium filling of the external iliac veins (*arrowheads*)

Among all 45 bodies, we found a total of 105 arterial (average 5.8, median 5) and 79 venous (average 4.2, median 5) lesions. Forty-one (91%) bodies had pelvic fractures. The total number of pelvic fractures in this group was 195. The details about the most injured vessels and pelvic bone fractures are shown in Table 2 *.* Ten arterial bleeding sites in seven different anatomical locations were significantly correlated with seven sites of pelvic fractures. Six venous bleeding sites in four different anatomical locations were significantly correlated with four pelvic fracture sites (Table 4 and Figs. 2 and 3). The numbers of arterial lesions per body were significantly associated with the severity of pelvic ring fracture according to Tile classifications (*p* = 0.012), unlike the number of venous bleeding sites (*p* = 0.34).

**Table 4:** Post-mortem cases—significant correlations between bleeding site and pelvic fracture

Pelvic vessels	Bone/articulation	*p* value
Arteries		
Right inferior gluteal artery	Right iliac wing	<0.05
Right iliolumbar artery	Right iliac wing	<0.05
Bilateral lateral sacral artery	Ipsilateral sacral wing	<0.05
Right superior gluteal artery	Right iliac wing	<0.05
Right superior gluteal artery	Right sacral wing	<0.05
Bilateral obturator artery	Ipsilateral iliopubic branch	<0.05
Bilateral obturator artery	Ipsilateral acetabulum	<0.05
Veins		
Left iliolumbar vein	Left sacroiliac disjunction	<0.05
Right lateral sacral vein	Right sacral wing	<0.05
Bilateral obturator vein	Ipsilateral iliopubic branch	<0.05
Bilateral obturator vein	Ipsilateral acetabulum	<0.05

Among our 45 post-mortem cases, there were four with such as an extensive pelvic bleeding that it was considered as the leading cause to death on the basis of the conventional autopsy following MPMCTA. All these four cases had a Tile C fracture, and the most frequent vascular injuries involved the obturator and superior gluteal arteries.

## Discussion

Our study showed that in blunt pelvic trauma, certain anatomical sites of arterial haemorrhage are associated with certain pelvic bone fractures. Indeed, in patients, we detected five correlations between arterial lesions and fractured pelvic bones (Table 3). In our post-mortem group, we observed seven artery-bone correlations. Moreover, MPMCTA revealed four associations between venous lesions and a fractured pelvic bone. To the best of our knowledge, anatomical correlations between bleeding sites and pelvic fractures have not been investigated previously in a consecutive clinical series of polytrauma patients neither in post-mortem cases. In the latter, Baqué et al. investigated the most frequent bleeding sites caused by pelvic open-book fractures [7]. The iliolumbar vessels were the most vulnerable pedicle in cases of sacroiliac joint disjunction [7] or iliac wing fracture [4] due to their anatomical relationship; this finding was confirmed by our statistically significant results.

In our patient group, the superior gluteal artery was the most frequently ruptured vessel, and there were also statistically significant associations between superior gluteal artery rupture and sacroiliac disjunction as well as sacral wing fracture, as previously reported [13, 24]. The second most frequently bleeding artery in our patients was the lateral sacral artery, which is typically injured after disruption of the posterior pelvic ring [13], including sacral wing fractures [4]; this finding was confirmed by our post-mortem results. Baqué et al. and Huittinen et al. considered the sacroiliac region the most important damage-prone pelvic area [6, 7], in agreement with our study, since sacral wing fracture was the most frequent type of pelvic fracture in both groups.

Additionally, bleeding of the lateral sacral artery occurred statistically more often in patients admitted for fall injuries, compared to patients presenting with traffic accidents or crushes. We explain this result by the mechanism inherent in most falls: The patients’ posterior pelvic ring is frequently the most vulnerable area of the body, since it is injured first and even with the highest force, when the patient after falling down hits the ground. In our post-mortem cases, we did not find this relationship, possibly due to the lower percentage of this mechanism of injury: only 11% (*n* = 24) of these cases were falls, unlike 49% (*n* = 24) in our polytrauma group.

The third most often injured artery in our patients was the obturator artery. This artery is known to typically bleed after acetabular or pubic rami fractures or symphysis disjunctions [4], which was again confirmed by our results.

Our study on post-mortem cases revealed that the number of injured arteries per body was significantly associated with the severity of pelvic ring fracture according to Tile classification. Despite a similar percentage of cases with pelvic fractures in both groups, we observed a higher number of Tile C fractures in the post-mortem cases than in living polytrauma patients (14 vs. 10) and, thus, a higher severity of pelvic fractures. This difference in fracture severity could explain the weaker association between vascular lesions and pelvic fractures we found in our patient group.

Using the Burgess and Young classification system for pelvic fractures, Dalal et al. and Magnussen et al. showed a clear association between the degree of pelvic ring disruption and vascular compromise [25] and consequent blood transfusion requirements [12]; however, they did not distinguish between venous and arterial bleeding. We used Tile classification instead of the classification system established by Young and Burgess. While both classifications differentiate between pelvic fractures according to the force vector that caused them, we think that the former is more straightforward and easier to apply [11, 20, 22].

Three types of bleeding may occur in severe pelvic fractures: arterial bleeding due to pelvic artery disruption, venous bleeding due to tearing or shearing of the pelvic veins and bleeding directly from fractured cancellous bone [5, 6, 8, 9]. In polytrauma patients, the haemodynamic consequences of venous bleeding after pelvic fractures are not well known, since they have been rarely reported. According to Baqué et al., in severe pelvic trauma, venous bleeding is more frequent than arterial haemorrhage, since venous walls are more fragile than the arterial walls. However, haemorrhages originating from venous dilacerations should be less serious than arterial bleeding because of the low blood pressure in the venous network. Due to pressure of the adjacent pelvic viscera, spontaneous haemostasis may occur [7].

In our group of polytrauma patients, we did not detect any actively bleeding veins, while we observed venous contrast extravasations in 35 out of our 45 post-mortem cases. The difference in the acquisition and injection parameters between our polytrauma MDCT and MPMCTA protocol is almost certainly responsible for this result, since we are convinced that venous lesions must have occurred in these severely injured patients. First, since the radiation exposure is not an issue when performing MPMCTA, unlike in polytrauma patients, we acquired four different acquisition phases in the post-mortem group. In our polytrauma patients, we performed a portovenous abdominopelvic acquisition only. Second, in cadavers, we used a far higher volume of contrast medium than in polytrauma patients [16, 19]. This difference may have had a definitive impact on the detection of arterial and venous lesions. Third, by using MPMCTA, the venous system is investigated separately from the arterial system. Injecting contrast media via a femoral venous cannula allows direct filling of the venous compartment without arterial filling. Finally, haemodynamically unstable polytrauma patients are in shock; therefore, their sympathetic nervous system is highly activated. Thus, consecutive vasoconstriction may prevent optimal contrast medium filling of the vessels and, consequently, decrease the degree of bleeding, seen as contrast media extravasation.

Comparing the diagnostic value of ante-mortem (MDCT) and post-mortem CT (MPMCTA) performed in eight trauma victims, Palmiere et al. reported higher sensitivity of MPMCTA for acute arterial or venous haemorrhage. Unfortunately, only one patient with pelvic injury was included [15] in this study.

Our study has several limitations, the most important of which is its retrospective design. The patients in the study had been investigated with a sole portovenous phase, according to our polytrauma protocol. Indeed, the portovenous or late phase has been proven to show contrast media extravasation with higher sensitivity and accuracy than the arterial phase [2, 9, 26–28]. However, an additional arterial phase might have enabled diagnosis of any simultaneous venous bleeding located adjacent to an injured artery that had been overlooked, since in the present study, it could not be distinguished from nearby arterial bleeding on the basis of the venous phase only [9, 28].

A second limitation is that several correlations between vessels and fractured bone were significant for one side of the pelvis but not for the contralateral side; this is due to the limited number of patients and post-mortem cases that were included. Larger investigations should confirm the results for the contralateral side.

A third limitation is that we compared two different techniques of acquisition, MDCT and MPMCTA, by including two different population groups. Ideally, we would have compared the same subjects in an ante- vs. post-mortem approach. However, in our practice, MPMCTA is only realised for forensic issues. Thus, extremely few subjects are investigated by MDCT before and by MPMCTA after death. Furthermore, only ten of our polytrauma patients died from their accident, and none of them due to pelvic bleeding. In our original database, there was only one case, in which both examinations were performed: MDCT before and MPMCTA after death. However, since MPMCTA was performed after arterial embolisation, we had to exclude this case according to our inclusion criteria. In our post-mortem group, pelvic bleeding sources were lethal in only three cases (7%).

Finally, one could argue that some of the vessel injuries detected in our post-mortem group may correspond to post-mortem changes. However, the rapid fatal outcome of polytrauma victims without prolonged agony makes contrast extravasation due to decomposition of the bodies, which naturally occurs with time, extremely unlikely [29, 30].

In conclusion, we found that in the presence of severe blunt trauma pelvic injury, certain arterial and venous bleeding sites were significantly correlated with underlying pelvic fracture locations. In post-mortem cases, the number of arterial lesions depended significantly on the severity of the pelvic fracture. Venous bleeding was only visible post-mortem, unlike in polytrauma patients, which we attribute to the difference in acquisition parameters used. Therefore, not only arterial bleeding but also venous lesions contribute to severe pelvic haemorrhage, supporting the hypothesis that the importance of pelvic venous bleeding is underestimated in patients. Future investigations should evaluate the proportional contribution of venous bleeding to overall pelvic haemorrhage as well as its clinical significance.
